# Role of Fenugreek in the prevention of type 2 diabetes mellitus in prediabetes

**DOI:** 10.1186/s40200-015-0208-4

**Published:** 2015-10-02

**Authors:** Arpana Gaddam, Chandrakala Galla, Sreenivas Thummisetti, Ravi Kumar Marikanty, Uma D. Palanisamy, Paturi V. Rao

**Affiliations:** Department of Endocrinology and Metabolism, Nizam’s Institute of Medical Sciences University, Punjagutta, Hyderabad, 500082 India; School of Medicine and Health Sciences, Monash University Malaysia, Selangor, Malaysia

**Keywords:** Fenugreek, Prediabetes, Impaired fasting glucose, Impaired glucose tolerance

## Abstract

**Background:**

It is hypothesized that dietary supplementation with Fenugreek modulates glucose homeostasis and potentially prevents diabetes mellitus in people with prediabetes. The objective of present study is to determine whether Fenugreek can prevent the outcome of T2DM in non diabetic people with prediabetes.

**Methods:**

A 3-year randomized, controlled, parallel study for efficacy of Fenugreek (*n* = 66) and matched controls (*n* = 74) was conducted in men and women aged 30–70 years with criteria of prediabetes. Fenugreek powder, 5 g twice a day before meals, was given to study subjects and progression of type 2 diabetes mellitus (T2DM) was monitored at baseline and every 3 months for the 3-year study.

**Results:**

By the end of intervention period, cumulative incidence rate of diabetes reduced significantly in Fenugreek group when compared to controls. The Fenugreek group also saw a significant reduction in fasting plasma glucose (FPG), postprandial plasma glucose (PPPG) and low density lipoprotein cholesterol (LDLc) whereas serum insulin increased significantly. It was observed that controls had 4.2 times higher chance of developing diabetes compared to subjects in the Fenugreek group. The outcome of diabetes in Fenugreek group was positively associated with serum insulin and negatively associated with insulin resistance (HOMA IR).

**Conclusions:**

Dietary supplementation of 10 g Fenugreek/day in prediabetes subjects was associated with lower conversion to diabetes with no adverse effects and beneficial possibly due to its decreased insulin resistance.

## Background

An epidemic of diabetes threatens the health of a large number of individuals in developed and developing countries alike [1]. Recent data from the USA indicate that the prevalence of prediabetes is 34.6 %, impaired fasting glucose (IFG) is 19.4 %, impaired glucose tolerance (IGT) is 5.4 % and that of IFG and IGT is 9.8 % in the adult population [2]. The epidemic of prediabetes is likely to compound the existing diabetes crisis as many individuals with IGT will develop type 2 diabetes mellitus (T2DM) in the future. It is estimated that approximately 316 million people worldwide are with IGT and this is predicted to rise to 471 million by 2035. According to the estimates of International Diabetes Federation (IDF) in 2013, India alone has 65.1 million people living with diabetes; this places India second to China [3]. Plants provide an excellent source of drugs and a large proportion of currently-available drugs have been either derived directly or indirectly from plant sources. Present literature suggests the existence of more than 800 plants that may possess hypoglycemic activity [4]. The use of medicinal plants in most developing countries, as a normative basis for the maintenance of good health, has been observed extensively. There is a growing global interest in herbal and other forms of traditional medicine [5].

An effective strategy to restrict global impacts of T2DM is by limiting the number of prediabetics [6]. It’s our focus to identify new effective therapeutic agents, with relatively low cost and low toxicity that can be used regularly to control a progression of T2DM in the prediabetic population. Thus, dietary supplements that can modulate glucose homeostasis and potentially improve lipid parameters would be desirable. Although numerous herbs are reported to possess antidiabetic activity, a significant amount of research and traditional usage suggests that Fenugreek seeds (*Trigonella foenumgraecum*) are among the best in terms of safety and efficacy [7]. Seeds of Fenugreek are a rich source of fiber and have multiple benefits in patients with diabetes [8]. Research in the past two decades has shown that Fenugreek seeds help to lower blood glucose in patients with diabetes. Its role as an antidiabetic, by reducing fasting blood glucose levels and improved glucose tolerance in human subjects was reported [9]. Fenugreek is currently available as a nutraceutical with claims to reduce hyperglycemia [10].

Patients, who already manifest abnormalities of glucose handling, could benefit from a low-risk, inexpensive, food-based intervention aimed at normalizing their metabolic milieu. From the literature it is observed that Fenugreek is a dietary supplement that may hold promise in this regard. Though the hypoglycemic and hypolipidemic effects of Fenugreek were evaluated in animal and human models with T2DM [11], no study has been reported in prediabetics. The present study assesses the ability of Fenugreek to prevent T2DM in prediabetics having IGT/IFG. The objective of present study is to determine whether Fenugreek can prevent the outcome of T2DM in non diabetic people with prediabetes. If pharmacotherapy for prediabetes is initiated, it is important to realize that any therapy will require a long-term commitment by both subject and provider. In addition, it is important to review with the patient that there are limited data demonstrating the long-term health benefits of pharmacological intervention compared with lifestyle intervention [12]. Therefore the present Fenugreek interventional study was carried out for a period of 3 years.

## Methods

The institutional ethics committee reviewed and approved the research protocol and the informed consent vide review letter No. IRB/NIMS/080/2010. It is also confirmed that the ethics committee of Nizam’s Institute of Medical Sciences is constituted and functions as per Good Clinical Practice guidelines issued by Central Drug Standard Control Organisation and Ethical guidelines for Biomedical research on Human subjects, issued by Indian Council of Medical Research.

### Selection of the subjects

The proposed study was carried out in the Diabetes Day Care Center of the University Department of Endocrinology and Metabolism at Nizam's Institute of Medical Sciences, Hyderabad. The research protocol and the informed consent was reviewed and approved by the Institutional Ethics Committee of Nizam’s Institute of Medical Sciences University, Hyderabad, India.

Men and women aged between 30–70 years with body mass index (BMI) ≥ 19 kg/m^2^, fasting plasma glucose (FPG) 100–125 mg/dl (IFG) (or) post 75 g oral glucose load, plasma glucose (oral glucose tolerance test, OGTT) 140–199 mg/dl (IGT) and those who were willing to give informed consent form were included in the study. Type 1 diabetes subjects, those who were taking drugs that could alter glucose tolerance, whose fasting triglycerides (TG) were >400 mg/dl, those who had a history of cancer or any major illness of the liver, kidney and central nervous system and women who were pregnant, breast feeding or planning a pregnancy during the course of the study were excluded from the study.

### Study design

**Fig. 1:**
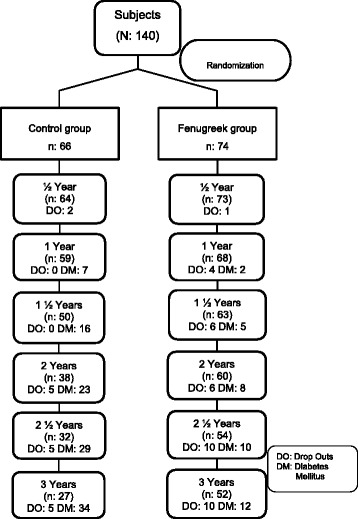
Subject disposition

A single blinded (subjects were blinded to allocation), 3-year follow-up of randomized controlled trial of Fenugreek in 66 control and 74 study (Fenugreek) subjects was initiated in nondiabetic people with prediabetes (Fig. 1 and Tables 1 and 2). The proposed study design included investigation of long-term intake of Fenugreek intervention in persons with prediabetes. Study subjects and matched-controls were selected and monitored once in every 3 months, for identifying study-specific changes during and after study period.

**Table 1:** Incidence rate of diabetes during the study period in controls

Control group							
	Baseline (*n* = 66)	½ yr (*n* = 64)	1 yr (*n* = 59)	1 ½ yrs (*n* = 50)	2 yrs (*n* = 38)	2 ½ yrs (*n* = 32)	3 yrs (*n* = 27)
NGT	0	11 (17.19)	16 (27.12)	15 (30.00)	9 (23.68)	11 (34.38)	5 (18.52)
IFG	23 (34.85)	14 (21.88)	11 (18.64)	4 (8.00)	4 (10.53)	6 (18.75)	5 (18.52)
IGT	16 (24.24)	6 (9.38)	10 (16.95)	13 (26.00)	10 (26.32)	7 (21.88)	5 (18.52)
IFG + IGT	27 (40.91)	26 (40.63)	13 (22.03)	11 (22.00)	9 (23.68)	3 (9.38)	10 (37.04)
No; for analysis	66	57	50	43	32	27	25
NDDM	0	7 (10.94)	9 (15.25)	7 (14.00)	6 (15.79)	5 (15.63)	2 (7.41)
Known DM	0	0	7	16	23	29	34
Drop outs	0	2	0	0	5	5	5

*NDDM* newly diagnosed diabetes mellitus, *NGT* normal glucose tolerance, *IFG* impaired fasting glucose, *IGT* impaired glucose tolerance, *DM* diabetes mellitus. NGT: FPG <100 and PPPG < 140 mg/dl, IFG: FPG 100–125 mg/dl, IGT 140–199 mg/dl, DM: FPG > 125 and PPPG > 200 mg/dl

Figures in parenthesis represent the percentage (%)

**Table 2:** Incidence rate of diabetes during the study period in Fenugreek group

Fenugreek group							
	Baseline (*n* = 74)	½ yr (*n* = 73)	1 yr (*n* = 68)	1 ½ yrs (*n* = 63)	2 yrs (*n* = 60)	2 ½ yrs (*n* = 54)	3 yrs (*n* = 52)
NGT	0	16 (21.92)	21 (30.88)	16 (25.40)	19 (31.67)	19 (35.19)	18 (34.62)
IFG	27 (36.49)	7 (9.59)	12 (17.65)	5 (7.94)	13 (21.67)	6 (11.11)	10 (19.23)
IGT	19 (25.68)	11 (15.07)	6 (8.82)	7 (11.11)	4 (6.67)	13 (24.07)	6 (11.54)
IFG + IGT	28 (37.84)	37 (50.68)	26 (38.24)	32 (50.79)	22 (36.67)	14 (25.93)	13 (25.00)
No; for analysis	74	71	65	60	58	52	47
NDDM	0	2 (2.74)	3 (4.41)	3 (4.76)	2 (33.33)	2 (3.70)	5 (9.62)
Known DM	0	0	2	5	8	10	12
Drop outs	0	1	4	6	6	10	10

*NDDM* newly diagnosed diabetes mellitus, *NGT* normal glucose tolerance, *IFG* impaired fasting glucose, *IGT* impaired glucose tolerance, *DM* diabetes mellitus. NGT: FPG <100 and PPPG < 140 mg/dl, IFG: FPG 100–125 mg/dl, IGT 140–199 mg/dl, DM: FPG > 125 and PPPG > 200 mg/dl

Figures in parenthesis represent the percentage (%)

All subjects in control as well as study groups were given similar instructions of physical activity and diet (adaptation of standard life style measures advised were, on dietary modification appropriate for weight and activity, weight reduction as necessary and exercise/physical activity for at least 30 minutes every day for a minimum of 5 days every week). Control and study subjects were counseled once in 3 months with advice on appropriate lifestyle and dietary practices.

### Randomization procedures

After steps of consenting, screening, and diet and lifestyle training, all subjects were randomly assigned to either the Fenugreek group or control group using a fixed randomization scheme with assignment based on computer-generated random numbers performed by an independent researcher. The allocation scheme was sealed in opaque and consecutively numbered envelopes. Envelopes were opened sequentially by the independent person.

### Fenugreek intervention program

The use of Fenugreek has been limited by its bitter taste and pungent odor. Isolation of biologically active components or production of a debitterized extract, which would allow greater use of the plant, has been investigated [13]. Debitterized, defatted and deodorized Fenugreek fiber with vitamins, minerals and amino acids was supplied (single batch) by SMS Pharmaceuticals limited (Jeedimetla, Hyderabad, India). The compound was in the form of a fine powder. Fenugreek powder (debitterized and processed) 5 g twice a day, was given to the subjects along with 200 ml of water half an hour before meals and they were asked to follow the same dosage regime up to the end of study. Number of Fenugreek packs supplied to and returned by the subjects at the follow-up visit was reported to calculate the compliance of study medication. Efficacy parameters were assessed at each visit.

### Data collection and measurable methods

Measurements were made at baseline (before treatment) and once in every 3 months during the study period. Demographic data was recorded at the baseline; a questionnaire on medical history and medication was administered, and body weight, height, and vital signs were measured. Height, weight, BMI, waist-to-hip ratio (WHR), FPG, post prandial plasma glucose (PPPG), lipid profile - serum cholesterol, serum triglycerides (TG), high density lipoprotein cholesterol (HDLc), low density lipoprotein cholesterol (LDLc) and serum insulin were recorded at baseline and during follow-up visits.

By measuring with tape horizontally, the WHR was calculated, waist as the minimal abdominal circumference located midway between the lower rib margin and the iliac crest and hip as the widest circumference over the great trochanters. The diagnosis of CAD (coronary artery disease), based on the presence of angina symptoms and abnormalities in resting electrocardiogram, was also assessed at baseline and after each year during the follow-up. Hypertension was determined by history of high blood pressure (≥130/85 mmHg).

Dyslipidemia was defined by any of the following: total cholesterol ≥200 mg/dl, triglycerides ≥150 mg/dl, HDL cholesterol ≤35 mg/dl, and/or LDL cholesterol ≥100 mg/dl or taking lipid-lowering drugs. OGTT at 2 h was performed in all subjects by taking 75 g oral glucose solution after overnight fasting; and then 2 h later, blood glucose level was measured. Blood was collected at 8:00 AM from the antecubital vein while the subjects were in the recumbent position after an overnight fasting. FPG, HbA1c, total cholesterol, triglyceride, HDLc, LDLc and serum insulin levels were measured according to the standard procedures and the sample analyses were done at Vimta Labs Ltd which is approved by the College of American Pathologists. HOMA-IR was calculated to assess change of IR.

### Statistical analysis

It was assumed that during a 3-year follow-up period, 36 % subjects with prediabetes (IFG or IGT) develop clinical diabetes, with annual incidence rates of conversion ranging between 10 and 12 % [14–16]. In the proposed 3-year study it is expected that administration of Fenugreek reduces the risk of diabetes development in 14 % study subjects. Assuming the risk errors of α = 0.05 and β = 0.20, sample size was calculated based on the normal approximation to the binominal and was found to be 73 subjects per group including 20 % drop outs during the study.

For analysis of outcome variables, values of mean (SD) at baseline and at the end of 3 years were presented for both the groups. Statistical analysis was performed by two-tailed paired *t*-test for numerical variables, chi-square test for ordinal variables as appropriate, multivariate analysis; significance was set at *p* < 0.05. Cumulative incidence rate of diabetes during the study period was calculated as the proportion of individuals who developed diabetes for every six months (E/N, number of events (E)/number of persons (N)). Relative risk reduction rate (RRR, control event rate―study event rate/control event rate) for developing diabetes was calculated at the end of 3 years. Insulin secretion and insulin sensitivity were calculated using a pre-validated formula, homeostasis model assessment (HOMA). Statistical analysis was performed using the Statistical Package for Social Sciences 13.0 software ((SPSS Inc., Chicago, IL).)

## Results

### Cumulative incidence rate and relative risk reduction of diabetes

**Fig. 2:**
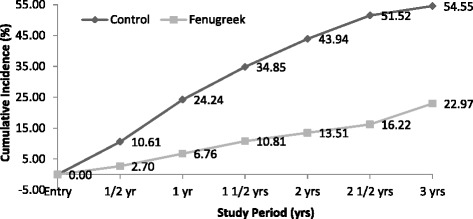
Cumulative Incidence Rate of Diabetes. During the study period at ½, 1, 1½, 2, 2½ and 3 years cumulative incidence rates in control and Fenugreek groups are represented

Cumulative incidence rates of diabetes at every six months for the length of 3 years were 10.6, 24.2, 34.9, 43.9, 51.5 and 55.0 % respectively in the control and 2.7, 6.7, 10.8, 13.5, 16.2 and 23.0 % respectively in the Fenugreek group (Fig. 2). By the end of the 3-year intervention period, the cumulative incidence rate of diabetes reduced significantly in Fenugreek group compared to the control (chi-square = 13.4; *p* < 0.01). RRR for developing diabetes at the end of 3 years was 0.6 in Fenugreek group.

### Anthropometric measurements

At baseline and at the end of 3 years, there were no significant differences in body weight, BMI, WHR, SBP and DBP (mean ± SD) within control and Fenugreek groups (Table 3).

**Table 3:** Mean anthropometric and biochemical measurements in control and Fenugreek groups during the study period

	Controls (*n* = 27)		Fenugreek (*n* = 52)	
	Baseline	3 years^a^	Baseline	3 years^a^
Weight, kg	68.53 (10.08)	68.34 (10.09)	69.13 (7.95)	68.79 (8.43)
BMI, kg/m^2^	25.95 (3.04)	25.91 (3.38)	26.62 (2.82)	26.43 (3.00)
WHR	0.92 (0.06)	0.91 (0.06)	0.92 (0.07)	0.92 (0.06)
SBP, mmHg	124.56 (14.89)	119.80 (24.66)	124.36 (18.80)	125.09 (18.58)
DBP, mmHg	80.72 (8.62)	77.72 (7.07)	79.36 (11.51)	80.36 (10.64)
FPG, mg/dl	102.8 (9.4)	100.6 (11.04)	103.7 (9.5)	99.7 (11.4)^*^
PPPG, mg/dl	146.1 (30.3)	147.3 (32.6)	142.9 (26.6)	129.0 (29.6)^**^
S.CHOL, mg/dl	185.5 (38.0)	179.9 (28.5)	183.1 (30.8)	183.9 (34.0)
S.TG, mg/dl	154.4 (78.1)	152.0 (50.6)	152.1 (73.0)	155.1 (69.6)
HDLc, mg/dl	36.5 (7.4)	38.1 (9.2)	37.9 (7.2)	36.4 (6.6)
LDLc, mg/dl	119.0 (28.3)	111.4 (23.3)	117.6 (26.3)	110.9 (23.9)^*^
S.Insulin, mU/l	12.2 (7.8)	10.9 (6.9)	10.2 (4.5)	12.0 (5.6)^**^
HOMA IR	3.2 (2.2)	2.7 (1.8)	2.6 (1.1)	3.0 (1.5)^*^

Values are presented in Mean (Standard Deviation); *BMI* body mass index, *WHR* waist to hip ratio, *SBP* systolic blood pressure, *DBP* diastolic blood pressure. Data were calculated by dependent sample *t* test

^a^No significant difference between baseline and 3 years study for both control and Fenugreek groups. Data were calculated by dependent sample *t* test. ^*^p < 0.05, ^**^p < 0.01: change from baseline within the control and Fenugreek groups. *FPG* fasting plasma glucose, *PPPG* post prandial plasma glucose, *S. CHOL* serum cholesterol, *S.TG* serum triglyceride, *HDLc* high density lipoprotein cholesterol, *LDLc* low density lipoprotein cholesterol, *VLDLc* very low density lipoprotein cholesterol, *S.Insulin* serum insulin, *HOMA IR* insulin resistance

### Biochemical parameters

#### Blood glucose

Subjects within control group had similar FPG and PPPG by the end of study period. In Fenugreek group, FPG (103.7 ± 9.5 vs 99.7 ± 11.4 mg/dl; *p* < 0.05) and PPPG (142.9 ± 26.6 vs. 129.0 ± 29.6 mg/dl; *p* < 0.01) had reduced significantly at the end of 3 years (Table 3).

### Lipid profile

When compared with baseline, serum cholesterol, TG, HDLc were comparable in controls and Fenugreek group at the end of study period.

However, LDLc was observed to reduce significantly at the end of 3 years in the Fenugreek group whilst this was not the case in the control group (117.6 ± 26.3 vs. 110.9 ± 23.9 mg/dl; *p* < 0.05) (Table 3).

### Serum insulin

Serum insulin levels and insulin resistance (HOMA IR) were similar in control group throughout the study period (Table 3). This was not the same in the Fenugreek group, both serum insulin levels (10.2 ± 4.5 vs. 12.0 ± 5.6 mU/l; *p* < 0.01) and HOMA IR (2.6 ± 1.1 vs. 2.9 ± 1.5; *p* < 0.05) increased significantly at the end of study period (Table 3).

### Multivariate analysis

Multiple regression analyses for associations between presence of diabetes and conventional risk variables in diabetes viz. gender (both men and women) (Odds Ratio, OR 0.96, 95 % Confidence Interval, CI 0.21–4.43), interventional groups (OR 4.18, 95 % CI 1.34–13.09; *p* < 0.05), age (30–70 years) (OR 1.05, 95 % CI 0.99–1.12), BMI (OR 0.96, 95 % CI 0.75–1.23), waist circumference (OR 0.97, 95 % CI 0.85–1.11), hip circumference (OR 1.00, 95 % CI 0.88–1.14), SBP (OR 0.98, 95 % CI 0.94–1.03), DBP (OR 1.00, 95 % CI 0.93–1.06), serum cholesterol (OR 1.00, 95 % CI 0.98–1.03), serum TG (OR 1.00, 95 % CI 0.99–1.01), HDLc (OR 0.97, 95 % CI 0.90–1.04), serum insulin (OR 1.98, 95 % CI 1.30–3.02; *p* < 0.01) and HOMA IR (OR 0.07, 95 % CI 0.02–0.30; *p* < 0.001) were estimated.

Parameters entered into the model showed a significant change in 2 log likelihood ratio (*p* < 0.001). The overall *p* value for the logistic regression analysis (*p* = 0.0002) indicates that the independent variables significantly predicts change in the outcome variable, which is onset of diabetes.

**Fig. 3:**
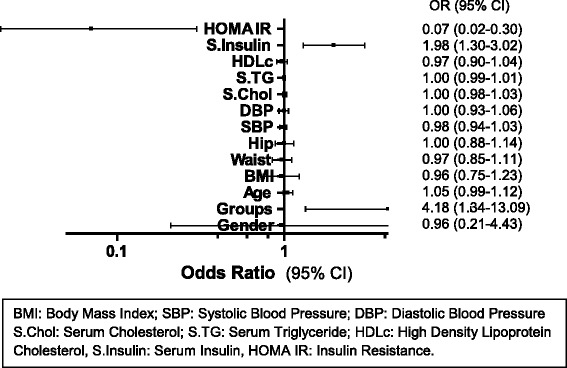
Forest plot for multivariate analysis between independent predictor variables and the outcome

The odds ratio of 4.18 (*p* = 0.01) for the intervention group indicates that subjects in control group have 4.2 times higher chance of developing diabetes when compared to the subjects in Fenugreek group. It was observed that the outcome of diabetes in Fenugreek group was positively associated with serum insulin (OR 1.98, 95 % CI 1.30–3.02; *p* < 0.01) and negatively associated with HOMA IR (OR 0.07, 95 % CI 0.02–0.30; *p* < 0.001) (Fig. 3 and Table 4). The results of regression analysis i.e., the individual odds ratio and their confidence intervals are represented graphically by Forest plot based on logistic regression (Fig. 3).

**Table 4:** Independent predictors of diabetes on Multinomial logistic regression at the end of the study (3 years)

Independent variables	B coefficient	Odds ratio	95 % CI		*p*- value
			Lower bound	Upper bound	
Gender	−0.04	0.96	0.21	4.43	ns
Groups	1.43	4.18	1.34	13.09	0.01^*^
Age, yrs	0.05	1.05	0.99	1.12	ns
BMI	−0.04	0.96	0.75	1.23	ns
Waist, cm	−0.03	0.97	0.85	1.11	ns
Hip, cm	0.00	1.00	0.88	1.14	ns
SBP, mmHg	−0.02	0.98	0.94	1.03	ns
DBP, mmHg	0.00	1.00	0.93	1.06	ns
S.CHOL, mg/dl	0.00	1.00	0.98	1.03	ns
S.TG, mg/dl	0.00	1.00	0.99	1.01	ns
HDLc, mg/dl	−0.03	0.97	0.90	1.04	ns
S.Insulin, mU/l	0.69	1.98	1.30	3.02	0.001^**^
HOMA IR	−2.67	0.07	0.02	0.30	0.0004^***^

*95* % *CI* 95 % confidence interval, *Groups* Control and Fenugreek groups, *S.CHOL* serum cholesterol, *S.TG* serum triglyceride, *HDLc* high density lipoprotein cholesterol, *S.Insulin* serum insulin, *HOMA IR* insulin resistance

^*^ *p* < 0.05, ^**^ *p* < 0.01, ^***^ *p* < 0.001

### Drug compliance

Drug compliance was calculated by pill count method at every follow-up visit as the percentage of difference between number of Fenugreek packs supplied and returned by the subject divided by the total study medication that subject has to take. Compliance varied at each follow-up visit with maximum number of subjects having good and acceptable compliance (Table 5).

**Table 5:** Compliance reported during the intervention period

Compliance	½ yr (n:73)		1 yr (n:68)		1 ½ yrs (n:63)		2 yrs (n:60)		2 ½ yrs (n:54)		3 yrs (n:52)	
	n	%	n	%	n	%	n	%	n	%	N	%
Poor	3	4.11	6	8.82	6	9.52	1	1.67	2	3.70	9	17.31
Average	8	10.96	7	10.29	3	4.76	5	8.33	9	16.67	7	13.46
Good and acceptable	62	84.93	55	80.88	54	85.71	54	90.00	43	79.63	36	69.23

If % of Drug Compliance: < 50 % - Non-Compliant, 50–70 % - Poor Compliant, 70–80 % - Average, > 80 % - Good and Acceptable

## Discussion

Globally, T2DM has an enormous impact on the health care costs and economy, as such it is highly desirable that this disease be prevented at an early stage. A prediabetes condition does not necessarily develop into diabetes if controlled well. Progression to diabetes can be prevented by lifestyle interventions and pharmacotherapeutic modes. There are several effective pharmacological regimens with encouraging results [17–19] however these regimens are either not usually economically accessible or not well tolerated by all [20]. Hence a need to focus on indigenous, inexpensive food-based regimens.

Traditional plant medicines are used throughout the world as alternative therapies to control diabetes. Although numerous herbs are reported to possess some degree of anti-diabetic activity [21], a significant amount of research, as well as traditional usage suggest that Fenugreek may be among the best in terms of efficacy and safety [7]. Though many studies evaluated the hypoglycemic and hypolipidemic effects of Fenugreek in animal and human models with type 1 and type 2 diabetes, no study is reported in prediabetes till date. Further this is a first study conducted employing a commercially debitterized Fenugreek powder in the prevention of diabetes in subjects with IFG/IGT. The rate of progression to diabetes during the intervention in controls and Fenugreek group is determined in the present study.

### Progression to diabetes

It was observed that the conversion rate from IFG and IGT to diabetes by the end of 3 years reduced significantly in Fenugreek group when compared to controls (Fig. 2). At 3 years, glucose levels have normalized in 18.52 % of controls and 34.62 % of Fenugreek subjects. In a 11-year follow up study it was stated that many people with prediabetes (a quarter or more) may revert to normal glucose tolerance on long term, and after a protracted follow-up, only about 50 % of people with IGT or IFG will develop diabetes [22]. In our study the conversion rate from IFG and IGT to diabetes in controls was similar to the study quoted above. Therefore this indicates that progression to diabetes from prediabetes stage is subsided by the consumption of Fenugreek as the conventional conversion rate of diabetes is lowered from 55 to 23 % at the end of 3 years due to Fenugreek.

### Anthropometric parameters

It was observed that body weight, BMI, SBP and DBP were unaltered in both control and Fenugreek groups. Simiarly the patient’s weight, BMI and other clinical parameters measured were found to be almost stationary when the dose-dependent effects of Fenugreek in diabetes with dyslipidemia were studied [23].

### Biochemical parameters

#### Hypoglycemic effects

During the study period FPG (*p* < 0.05) and PPPG (*p* < 0.01) reduced significantly at the end of 3 years in Fenugreek group.

The hypoglycemic effect of Fenugreek seed powder discussed in our study is well supported by few studies [9, 11, 24, 25] which showed hypoglycemic effect in both type 1 and type 2 diabetes subjects. *In vivo* study by Kumar RV et al. [26] depicted a significant reduction (*p* < 0.001) in the postprandial blood glucose levels in the diabetic rats on treatment with Fenugreek formulation AF40.

#### Hypolipidemic effects

In this study, serum cholesterol and TG levels were almost similar by the end of study period within control and Fenugreek groups, which is in contrast to the studies which reported that cholesterol and TG levels were lower in Fenugreek treated animals over untreated diabetic animals (*p* < 0.05) [27, 28]. The small changes in serum cholesterol and TG levels could be due to the fact that the mean data for these variables are already in the normal range.

Within controls and Fenugreek group there were no significant changes in HDLc throughout the study period. Analogous to these results few studies also reported that Fenugreek did not alter HDLc levels [23, 29]. The LDLc reduced at the end of 3 years (*p* < 0.05) in Fenugreek group. Fenugreek seeds contain a gel-like soluble fiber which combines with bile acid and lowers triglyceride and LDL cholesterol levels [30].

#### Insulinotropic effects

In controls, serum insulin levels remained same during the study period whereas in Fenugreek group, at the end of 3 years, serum insulin had increased significantly (*p* < 0.01) in Fenugreek group. Various studies [31, 32] suggested that Fenugreek seeds act as insulin secretor, as they reported increased insulin secretion in animal studies. Yadav et al. [33] also suggested that Fenugreek seeds, more precisely the water extract, act as an insulin secretor but they did not monitor insulin levels.

As serum insulin levels have increased in Fenugreek subjects, insulin resistance was assessed by HOMA. Insulin resistance (HOMA IR) was not different in control group during the intervention period but within Fenugreek group HOMA IR increased significantly at the end of 3 years (*p* < 0.05). This is in contrary with the study which reported that the action of Fenugreek is mediated by improving insulin sensitivity and decreasing insulin resistance apart from the known mechanisms of reduced glucose absorption [11]. The data obtained on insulin resistance in our study are not in agreement with the study where anti-diabetic properties of 4-hydroxy isoleucine, the active compound in Fenugreek were seen for its insulinotropic action and for extrapancreatic insulin-sensitizing effects [34]. The studies reported above were done in type 2 diabetes subjects and it cannot be assumed that Fenugreek actually increases insulin resistance in prediabetes, because the serum insulin levels in our study subjects were within the normal range. In addition, the HOMA-IR level in the Fenugreek group at the end of 3 years was within the normal insulin resistance category (<3). Regression analysis outcome of diabetes in Fenugreek group was positively associated with serum insulin (*p* < 0.01) and negatively associated with HOMA IR (*p* < 0.001). Fenugreek may exert its therapeutic effect through its alkaloids content by modulation of insulin secretion. The amino acid 2S,3R,4S, 4-hydroxyisoleucine, purified from Fenugreek seeds, showed insulinotropic effects which increased peripheral glucose uptake *in vitro* [35].

### Strengths, limitations and avenues of the study for future research

This is a prospective, randomized controlled study conducted in men and women, having different life-styles and socio-cultural backgrounds but satisfying the inclusion and exclusion criteria mentioned. Though the sample size had diversified demographics, their anthropometric, clinical and biochemical parameters were similar at baseline. This study could target large populations more cost effectively. So far there was no systematic long term study in prediabetes and as such this is a conventional intervention, carried out for the first time in a different group of population (prediabetes). No study until now has reported the incidence conversion rate to diabetes with the interventional Fenugreek powder in prediabetes. The strength of this intervention lies on the complexity of data analysis which illuminates the statistical correlation between independent risk factors towards the onset or progression to diabetes.

Our estimate of the effect of the intervention can be considered conservative for two reasons. First, data was analyzed according to the intention-to-prevent principle, even though some subjects in the intervention group did not follow the recommendations about Fenugreek consumption. Second, for ethical reasons, all subjects assigned to the control group also received general health advice and counseling on dietary patterns at base line and at annual follow-up visits and may have benefited from this advice.

Participants who dropped out before the end of the 1 year intervention period were not included in the analysis because if their last observation had been carried forward, the differences between the two groups would have been artificially maintained over the follow-up period. Carrying the last observation forward assumes that assigning a no-change status is a conservative analysis, which is not the case, because weight change over time is nonlinear.

Despite of supplying debitterized processed Fenugreek powder to the study group, few subjects have skipped their doses due to its unacceptable palatability. Because of this reason they were advised to consume it along with some flavoring agents. This perhaps, could alter the plasma glucose levels and was found to be one of the limitations of the study. Though there were many reasons for subject dropouts, one of the reasons was their unwillingness towards the consumption of Fenugreek on long term basis due to its undesirable taste. In addition, subjects did not receive any incentives during the study due to which there was a lost to follow-up and the dropout rate in study group was recorded as 13.5 %.

An alternative prediabetic intervention study can be carried out with the supplementation of tulsi with Fenugreek, which can be used to mask the bitter taste of Fenugreek. In addition, the role of Fenugreek in different population groups should also be carried out.

## Conclusions

This study provides evidence for the use of Fenugreek to delay the onset of diabetes in subjects with prediabetes. Fenugreek powder is useful to lower the blood glucose in prediabetes. From the results it can be concluded that Fenugreek showed hypocholesterolemic effects by reducing LDLc levels but without affecting serum TG, HDLc levels. Our results strongly suggest that the enhancement of serum insulin levels is due to insulinotropic effects. In conclusion, our results show that hypoglycemic effects are due to increasing levels of serum insulin and we suggest here that the mode of action of Fenugreek may be caused by their contents of alkaloids.

## Abbreviations

BMI: Body mass index
CI: Confidence interval
DBP: Diastolic blood pressure
FPG: Fasting plasma glucose
HDLc: High density lipoprotein cholesterol
HOMA: Homeostasis model assessment
ICMR: Indian Council of Medical Research
IDF: International Diabetes Federation
IFG: Impaired fasting glucose
IGT: Impaired glucose tolerance
LDLc: Low density lipoprotein cholesterol
OGTT: Oral glucose tolerance test
OR: Odds ratio
PPPG: Post prandial plasma glucose
RRR: Relative risk reduction
SBP: Systolic blood pressure
TG: Triglycerides
VLDLc: Very low density lipoprotein cholesterol
WHR: Waist to hip ratio
